# The opinions of migrant women from Ukraine regarding intrapartum and midwifery care in Poland: a cross-sectional comparative study

**DOI:** 10.3389/fpubh.2026.1845420

**Published:** 2026-07-29

**Authors:** Karolina Omachel, Saskia van den Bercken, Jakub Winkler-Galicki, Patryk Rzońca, Ewa Rzońca

**Affiliations:** 1 St. Sophia's Specialist Hospital, Żelazna Medical Center, Warsaw, Poland; 2 Department of Human Anatomy, Faculty of Health Sciences, Medical University of Warsaw, Warsaw, Poland; 3 Department of Obstetrics and Gynecology Didactics, Faculty of Health Sciences, Medical University of Warsaw, Warsaw, Poland

**Keywords:** childbirth, communication, discrimination, midwife, migrants, Poland, Ukraine, women

## Abstract

**Introduction:**

Since the escalation of the armed conflict in Ukraine in 2022, the number of Ukrainian citizens in Poland has increased considerably, especially among women. The study aimed to explore the opinions of migrant women from Ukraine regarding the intrapartum and midwifery care they received in Poland, as well as to analyse demographic, migration-related, clinical, and psychosocial variables associated with these aspects of care.

**Methods:**

The study was conducted using the diagnostic survey method between December 2024 and February 2025 among 481 migrant women from Ukraine who gave birth in Poland. The research tool was an original questionnaire developed in Ukrainian.

**Results:**

The migrant women from Ukraine reported high assessment scores for the intrapartum care (M = 8.8) and midwifery care (M = 9.0) they received in Poland. The variable most strongly associated with higher ratings of intrapartum and midwifery care was the fulfilment of childbirth related expectations (*p* < 0.05). A negative association with the assessment of intrapartum and midwifery care was observed for the experience of discrimination or humiliation, as well as for poorer treatment based on national origin (*p* < 0.05). In turn, a significant interaction effect was found between the fulfilment of childbirth-related expectations and the time of arrival in Poland, both for the overall assessment of intrapartum care (*β* = 0.328; *p* = 0.002) and for the assessment of midwifery care (*β* = 0.248; *p* = 0.021).

**Conclusion:**

The study underscores that the fulfilment of birth-related expectations, access to information regarding the medical situation, and the absence of discrimination or humiliation were associated with more favourable evaluations of intrapartum and midwifery care among migrant women from Ukraine in Poland.

## 1 Introduction

Human migration is now a common global phenomenon. It is one of the key components of the social, economic, and demographic transformations occurring in numerous countries. In Poland, the migration landscape changed significantly following the outbreak of the full scale war in Ukraine on 24 February, 2022. This resulted in a sudden influx of Ukrainian citizens into Poland. According to the report of the Office for Foreigners, the number of Ukrainian citizens with valid residence permits in Poland increased from 41,000 to 1,565,000 between 2014 and June 2025. It is worth emphasising that this is the largest group of foreign nationals, accounting for as much as 78% of all migrants in Poland. As of 1 July, 2025, the number of Ukrainian citizens under temporary protection amounts to 988,654 individuals. Among adults, about 72% are women (1). Such a rapid increase in the number of migrant women constitutes a new challenge for the healthcare system. One of the more difficult decisions for women of reproductive age is giving birth in another country. Consequently, perinatal care becomes a key component of the healthcare system (2).

The primary legal act regulating care for pregnant women, women in labour, women in the postpartum period, and newborns in Poland is the Organisational Standard of Perinatal Care. This document is based on the guidelines of the World Health Organisation, which emphasises that the perinatal period should constitute a positive experience (3–5). Legal acts regulating perinatal care in Poland underscore the need to tailor healthcare services to the individual needs of every woman. These documents also highlight the importance of person-centred treatment of patients (4–6).

The perinatal care system in Ukraine, unlike the Polish one, is not based on a single legal act. It is regulated by a set of smaller documents that compile the most essential information on specific aspects of care during pregnancy, childbirth, and the postpartum period. Nevertheless, all of them are based on standards set by the WHO, which means that perinatal care does not differ significantly between the two countries. An important difference between the Ukrainian and Polish systems is the absence of a unified document regulating the midwifery profession, which significantly diminishes the midwife’s role in caring for pregnant patients, during childbirth, and in the postpartum period (6–8).

Ukrainian refugee women struggle not only with differences between the healthcare systems, but also with the impact of the armed conflict ongoing in their home country. War related migration can cause stress and anxiety, which during the perinatal period may have adverse health consequences for both the woman and the child (9, 10). Existing literature reports a higher proportion of both preterm births and post term pregnancies among refugee women. There is also an increase in postpartum complications, along with higher rates of neonatal morbidity and mortality (10). A Ukrainian study examining the characteristics of perinatal care during martial law indicates a rising incidence of complications such as preeclampsia, anaemia, preterm birth, perinatal hypoxia, intracranial haemorrhage in newborns, jaundice, infections, and birth trauma. The authors of that study also observed an increase in the occurrence of genetic abnormalities in foetuses (11). The mental health of women living abroad during the perinatal period has also become an important area of research. Researchers point to a higher risk of anxiety and depressive disorders among this population. Their main causes are separation from family, feelings of isolation and loneliness, exposure to stress, and anxiety about their own health and the baby’s health (9, 12). Another significant stress inducing factor among migrant women from Ukraine is the growing proportion of individuals who express negative attitudes toward this minority group. In the period immediately following the outbreak of the war in Ukraine, the massive influx of refugees prompted a strong surge of public willingness to help. However, as the war continued, this initial tendency gradually declined, whereas negative attitudes toward refugees have shown a steady increase (13, 14).

The quality of perinatal care delivered to migrant women is also shaped by multiple other factors. Previous research has identified communication and the ability to exchange information effectively as key factors associated with migrant women’s experiences of maternity care (2, 15–21). In particular, women’s awareness, understanding, and ability to navigate the scope and availability of perinatal care services appear to be closely related to the quality of communication with healthcare professionals (2, 15–18, 21). Another relevant dimension is women’s sense of safety within the healthcare setting. Experiences of humiliation, discrimination, or perceived unequal treatment may adversely affect migrant women’s perceptions and evaluations of the care received (2, 19, 20, 22–24). Although intrapartum care in Poland is delivered by a multidisciplinary team, midwives play a central role in the care of women in an uncomplicated pregnancy and during childbirth, providing direct support, facilitating communication, and ensuring continuity of care. Therefore, this cross-sectional study aimed to explore the opinions of migrant women from Ukraine regarding the intrapartum and midwifery care they received in Poland, as well as to analyse demographic, migration-related, clinical, and psychosocial variables associated with these aspects of care, and identify areas for improving respectful, patient-centred maternity care for women from Ukraine in the context of war-related migration.

## 2 Methods

### 2.1 Study design and participants

A comparative cross-sectional survey was carried out between December 2024 and February 2025 among migrant women from Ukraine who had given birth in Poland. For comparative analyses, the timing of arrival in Poland served as the grouping variable: participants were classified as having arrived either before the outbreak of the full-scale war in Ukraine on 24 February 2022 or during the war. The inclusion criteria for the study were: age above 18 years, Ukrainian nationality, having given birth in Poland (most recent childbirth), and providing informed consent to participate in the study. The study excluded migrant women of non Ukrainian origin, individuals under the age of 18, women in their first pregnancy who had not yet given birth in Poland, and those who did not consent to participate. The time elapsed since the most recent childbirth in Poland was not used as an eligibility criterion but was recorded and subsequently analysed to assess the potential influence of recall bias. The study yielded 490 completed questionnaires, of which 9 were excluded based on the established criteria. Ultimately, 481 questionnaires were analysed.

### 2.2 Measures

The research tool was an original questionnaire developed specifically for the purposes of this study by the research team, based on a review of the literature, clinical experience, and the study objectives, and subsequently translated into Ukrainian. Before the main study, a pilot study was conducted among five Ukrainian-speaking migrant women to assess the comprehensibility of the questions and the linguistic appropriateness of the questionnaire. Based on the feedback received, minor editorial and linguistic revisions were made to the original instrument; however, it was not subjected to formal psychometric validation. The main study was distributed through social media forums focused on women of Ukrainian origin. The original questionnaire included questions on demographic and migration-related aspects (age, education, time of arrival in Poland – before the outbreak of the war vs. during the war, independent migration (as opposed to accompanied migration), and the ability to communicate in the Polish language); health status, including childbirth-related factors (e.g., labour complications, non pharmacological pain relief methods, epidural anaesthesia, episiotomy, labour induction, date of the most recent childbirth in Poland and preterm birth); as well as questions concerning the women’s experiences with intrapartum care in Poland (including: overall assessment of care, evaluation of midwifery care, fulfilment of childbirth-related expectations, being informed about the medical situation, experience of discrimination or humiliation, treatment based on national origin, and language barrier). Overall assessments of intrapartum and midwifery care were obtained using single numerical ratings to provide a brief, separate evaluation of women’s global perceptions of these two domains of care. This approach was selected to ensure the feasibility of the online questionnaire whilst allowing for the inclusion of a broad range of demographic, migration-related, clinical, and psychosocial variables.

### 2.3 Data analysis

Statistical analyses were conducted to identify factors associated with the assessment of intrapartum care and midwifery care in Poland among migrant women from Ukraine. In the first stage, a descriptive analysis of demographic, migration related, clinical, and psychosocial variables was performed, taking into account the time of arrival in Poland (before the outbreak of the war vs. during the war). Continuous variables with a non parametric distribution were presented as medians with interquartile ranges (Mdn [IQR]), whereas variables showing near normal distribution were presented as means with standard deviations (M ± SD). Qualitative variables were presented as numbers and percentages. Inter-group comparisons were performed using non parametric or parametric tests, depending on the data: the Mann–Whitney *U* test for continuous variables with a non parametric distribution, and the chi square (*χ*^2^) test for qualitative variables. A significance level of *p* < 0.05 was adopted in all analyses.

In the next stage, hierarchical linear regression analyses were conducted for both dependent variables. Independent variables were entered into the models in blocks, in accordance with the adopted theoretical assumptions. Model 1 included demographic and migration-related variables: age, education, arrival in Poland during the war, independent arrival, and the ability to communicate in the Polish language. Model 2 was expanded by adding clinical variables related to the course of childbirth, including complications, non-pharmacological pain relief methods, epidural anaesthesia, episiotomy, labour induction, term of delivery and, specifically in the analysis of midwifery care assessment, the mode of delivery by caesarean section. Model 3 included psychosocial predictors, namely the fulfilment of childbirth-related expectations, being informed about the medical situation, experience of discrimination or humiliation, treatment based on national origin, and language barrier. For each model, standardised regression coefficients (*β*), the coefficient of determination (*R*^2^), the adjusted coefficient of determination (adj. *R*^2^), and the change in explained variance (Δ*R*^2^) were calculated. Statistical inference for the hierarchical regression analyses was based on heteroscedasticity-consistent HC3 robust standard errors.

Before interpreting the final hierarchical regression models, diagnostic procedures were performed. Multicollinearity was assessed using variance inflation factors (VIFs) and tolerance values. Linearity and homoscedasticity were evaluated using residual-versus-fitted plots, whereas the distribution of residuals was assessed using Q–Q plots, histograms, and the Shapiro–Wilk test. Influential observations were examined using Cook’s distance, leverage values, and studentised residuals. Multicollinearity was not a concern in the final models, with VIF values below 1.60 and tolerance values above 0.63. Residual diagnostics indicated heteroscedasticity and deviations from normality; therefore, heteroscedasticity-consistent HC3 robust standard errors were used for statistical inference. No observations were excluded solely based on diagnostic criteria, as no individual case had a Cook’s distance approaching 1.

In the final stage, moderation analyses were conducted to determine whether the relationship between selected psychosocial predictors and the assessment of intrapartum care and midwifery care differed depending on the time of arrival in Poland. The moderator was the time of arrival in Poland (before the outbreak of the war vs. during the war). The models included both main effects and interaction terms (predictor × moderator). Moderation analyses were conducted separately for both dependent variables.

### 2.4 Ethical consideration

The research was conducted in accordance with the principles of the Helsinki Declaration, and the research project was acknowledged by the Bioethical Committee at the Medical University of Warsaw (AKBE/323/2024). Respondents were informed that participation was voluntary, and that study results were anonymous and to be used exclusively for research purposes.

## 3 Results

### 3.1 Characteristics of the study group

Table 1 presents selected demographic, migration-related, clinical, and psychosocial variables pertaining to the participants, grouped by the time of their arrival in Poland (before the outbreak of the war vs. during the war). A greater percentage of women with higher education was recorded among those who came to Poland during the war (81.2% vs. 71.1%, *p* < 0.05). Women who came to Poland before the war were significantly more likely to communicate in Polish (30.8% vs. 16.2%, *p* < 0.05) and to have arrived in Poland independently (30.8% vs. 16.2%, *p* < 0.05). Women who migrated to Poland during the war more frequently reported the availability of a Ukrainian-speaking person in the hospital (32.0% vs. 10.3%, *p* < 0.05) and the presence of language barriers (49.6% vs. 12.3%, *p* < 0.05). There were no statistically significant differences between the groups with respect to age or most clinical variables associated with the course of pregnancy and childbirth (*p* > 0.05).

**Table 1:** Demographic, clinical, and psychosocial characteristics of the study group according to the time of arrival in Poland.

Variables	Total	Arrival before the war	Arrival during the war	*p*-value
Demographic and migration-related characteristics				
Age – Mdn (IQR)	30 (27–34)	31 (27–34)	30 (27–34)	0.188
Education – *n* (%)				
Vocational	64 (13.3)	42 (16.6)	22 (9.6)	0.031
Secondary	52 (10.8)	31 (12.3)	21 (9.2)	
Higher	365 (75.9)	180 (71.1)	185 (81.2)	
Ability to communicate in Polish – *n* (%)	115 (23.9)	78 (30.8)	37 (16.2)	<0.001
Independent arrival in Poland – *n* (%)	115 (23.9)	78 (30.8)	37 (16.2)	<0.001
Clinical variables				
Number of pregnancies – *n* (%)				
1. Pregnancy	260 (54.1)	142 (56.1)	118 (51.8)	0.367
2. Pregnancies	131 (27.2)	62 (24.5)	69 (30.2)	
3. Or more pregnancies	90 (18.7)	49 (19.4)	41 (18.0)	
Number of births – *n* (%)				
1. Birth	315 (65.5)	176 (69.6)	139 (61.0)	0.132
2. Births	125 (26.0)	57 (22.5)	68 (29.8)	
3. Or more births	41 (8.5)	20 (7.9)	21 (9.2)	
Time since most recent childbirth in Poland, months – Mdn (IQR)	9 (5–21)	9 (5–23)	9 (5–17)	0.140
Complications in the last pregnancy – *n* (%)	137 (28.5)	70 (27.7)	67 (29.4)	0.677
Complications during childbirth – *n* (%)	68 (14.1)	30 (11.9)	38 (16.7)	0.131
Non-pharmacological pain-relief methods – *n* (%)	265 (55.1)	147 (58.1)	118 (51.8)	0.162
Vaginal delivery – *n* (%)	314 (65.3)	169 (66.8)	145 (63.6)	0.461
Labour induction – *n* (%)	196 (40.7)	100 (39.5)	96 (42.1)	0.565
Term birth – *n* (%)	451 (93.8)	237 (93.7)	214 (93.9)	0.934
Episiotomy – *n* (%)	91 (18.9)	47 (18.6)	44 (19.3)	0.840
Epidural anaesthesia – *n* (%)	251 (52.2)	136 (53.8)	115 (50.4)	0.467
Presence of a companion during childbirth – *n* (%)	368 (76.5)	197 (77.9)	171 (75.0)	0.459
Psychosocial variables				
Expectations during childbirth – *n* (%)				
Not fulfilled	46 (10.2)	25 (10.6)	21 (9.8)	0.729
Partially fulfilled	101 (22.5)	56 (23.7)	45 (21.0)	
Entirely fulfilled	303 (67.3)	155 (65.7)	148 (69.2)	
Being informed about the medical situation – *n* (%)	425 (88.4)	219 (86.6)	206 (90.4)	0.196
Experience of discrimination / humiliation – *n* (%)	46 (9.6)	26 (10.3)	20 (8.8)	0.575
Treatment based on national origin – *n* (%)				
Yes – worse	50 (10.4)	25 (9.9)	25 (11.0)	0.927
No	416 (86.5)	220 (87.0)	196 (85.9)	
Yes – better	15 (3.1)	8 (3.2)	7 (3.1)	
Unrealistic expectations – *n* (%)	33 (6.9)	16 (6.3)	17 (7.5)	0.624
Presence of a Ukrainian-speaking person in the hospital – *n* (%)	99 (20.6)	26 (10.3)	73 (32.0)	<0.001
Language barrier – *n* (%)	144 (29.9)	31 (12.3)	113 (49.6)	<0.001
Assessment of intrapartum care in Ukraine – M (SD)	7.3 (2.3)	6.9 (2.7)	7.6 (1.9)	0.374
Assessment of intrapartum care in Poland – M (SD)	8.8 (1.5)	8.8 (1.4)	8.8 (1.6)	0.688
Assessment of midwifery care during childbirth – M (SD)	9.0 (1.6)	9.1 (1.5)	9.0 (1.7)	0.882

### 3.2 The assessment of intrapartum care in Poland among migrant women from Ukraine

The results of the hierarchical linear regression analysis are presented in Table 2. Statistical inference was based on heteroscedasticity-consistent HC3 robust standard errors. Model 1, which included demographic and migration-related variables, did not explain a significant proportion of the variance in the assessment of intrapartum care (*R*^2^ = 0.006; adj. *R*^2^ = −0.005; robust omnibus Wald test, *p* > 0.05). After clinical variables were incorporated into Model 2, the explained variance increased by 0.078 (*R*^2^ = 0.084; adjusted *R*^2^ = 0.061). The added clinical block was statistically significant according to the robust Wald test [*F*(6, 438) = 3.849; *p* < 0.001]. In this model, complications during childbirth were associated with lower assessments of intrapartum care (*β* = −0.193; *p* < 0.01), whereas the use of non-pharmacological pain-relief methods was associated with more favourable assessments (*β* = 0.198; *p* < 0.001). After psychosocial variables were added in Model 3, the explained variance increased by a further 0.463 *R*^2^ = 0.549; adjusted *R*^2^ = 0.528, and Δ*R*^2^ = 0.465. The added psychosocial block was statistically significant according to the robust Wald test [*F*(6, 432) = 34.559; *p* < 0.001]. The strongest positive association was observed for fulfilment of childbirth-related expectations (*β* = 0.463; *p* < 0.001). Being informed about the medical situation was also significantly associated with a better assessment of care (*β* = 0.146; *p* < 0.001). A negative association with the assessment of intrapartum care was observed for the experience of discrimination or humiliation (*β* = −0.114; *p* < 0.01) as well as for worse treatment based on national origin (*β* = −0.166; *p* < 0.001). The ability to communicate in Polish remained positively associated with intrapartum care assessment in the final model (*β* = 0.083; *p* < 0.01).

**Table 2:** Hierarchical linear regression analysis for variables associated with the assessment of intrapartum care in Poland among migrant women from Ukraine.

Predictors	Model 1 *β*	Model 2 *β*	Model 3 *β*
Demographic and migration-related variables			
Age	0.041	0.058	0.034
Education	−0.029	−0.035	−0.007
Arrival in Poland during the war	0.013	0.042	−0.012
Independent arrival in Poland	0.018	0.012	−0.007
Ability to communicate in Polish	0.057	0.051	0.083*
Clinical variables			
Complications during childbirth	–	−0.193***	−0.053
Non-pharmacological pain-relief methods	–	0.198***	0.055
Epidural anaesthesia	–	0.020	−0.028
Episiotomy	–	−0.058	−0.007
Labour induction	–	−0.028	0.004
Term of delivery—full-term	–	0.010	0.012
Psychosocial variables			
Fulfilment of childbirth-related expectations	–	–	0.463***
Being informed about the medical situation	–	–	0.146***
Experience of discrimination/humiliation	–	–	−0.114**
Experience of worse treatment due to national origin	–	–	−0.166***
Experience of better treatment due to national origin	–	–	0.034
Language barrier	–	–	−0.018
*R* ^2^	0.006	0.084	0.549
*R*^2^ adj	−0.005	0.061	0.528
Δ*R*^2^	–	0.078***	0.465*
Robust Wald test for the added block		3.849***	34.559***
Robust omnibus Wald test	0.681	2.971***	14.998***

*β* = standardised regression coefficient. *p*-values were calculated using heteroscedasticity-consistent. HC3 robust standard errors. *p < 0.05, **p < 0.01; ***p < 0.001.

### 3.3 The assessment of midwifery care in Poland among migrant women from Ukraine

The results of the hierarchical linear regression analysis pertaining to midwifery care are presented in Table 3. Statistical inference was based on heteroscedasticity-consistent HC3 robust standard errors. Model 1, which included only demographic and migration-related variables, did not explain a significant proportion of the variance in the dependent variable (*R*^2^ = 0.005; adj. *R*^2^ = −0.006; robust omnibus Wald test, *p* > 0.05). After clinical variables were incorporated into Model 2, the explained variance increased by 0.103 (*R*^2^ = 0.108; adjusted *R*^2^ = 0.084). The added clinical block was statistically significant according to the robust Wald test [*F*(7, 437) = 5.359; *p* < 0.001]. At this stage of the analysis, complications during childbirth were associated with lower assessment scores for midwifery care (*β* = −0.162; *p* < 0.05), whereas the use of non-pharmacological pain-relief methods was associated with more favourable assessments (*β* = 0.241; *p* < 0.001). Episiotomy and epidural anaesthesia were not significantly associated with midwifery care assessment in Model 2. After psychosocial variables were added in Model 3, the explained variance increased by a further 0.329 (*R*^2^ = 0.437; adjusted R^2^ = 0.414). The added psychosocial block was statistically significant according to the robust Wald test [*F*(6, 431) = 16.109; *p* < 0.001]. The strongest positive association was the fulfilment of childbirth related expectations (*β* = 0.364; *p* < 0.001). Being informed about the medical situation was also associated with more favourable assessments of midwifery care (*β* = 0.176; *p* < 0.01). Negative associations were observed for experiences of discrimination or humiliation (*β* = −0.166; *p* < 0.01) and epidural anaesthesia (*β* = −0.105; *p* < 0.05). In addition, the use of non-pharmacological pain-relief methods remained positively associated with midwifery care assessment in the final model (*β* = 0.130; *p* < 0.01). Worse treatment based on national origin was not significantly associated with midwifery care assessment in the final model.

**Table 3:** Hierarchical linear regression analysis for variables associated with the assessment of midwifery care in Poland among migrant women from Ukraine.

Predictors	Model 1 *β*	Model 2 *β*	Model 3 *β*
Demographic and migration-related variables			
Age	0.016	0.026	0.008
Education	0.006	0.005	0.023
Arrival in Poland during the war	−0.018	0.007	−0.035
Independent arrival in Poland	0.043	0.037	0.018
Ability to communicate in Polish	0.039	0.032	0.060
Clinical variables			
Complications during childbirth	–	−0.162*	−0.041
Non-pharmacological pain-relief methods	–	0.241***	0.130**
Epidural anaesthesia	–	−0.056	−0.105*
Episiotomy	–	−0.106	−0.052
Labour induction	–	0.014	0.041
Term of delivery - full-term	–	0.041	0.036
Delivery by CC	–	−0.062	−0.026
Psychosocial variables			
Fulfilment of childbirth-related expectations	–	–	0.364***
Being informed about the medical situation	–	–	0.176**
Experience of discrimination / humiliation	–	–	−0.166**
Experience of worse treatment due to national origin	–	–	−0.094
Experience of better treatment due to national origin	–	–	0.026
Language barrier	–	–	−0.017
*R* ^2^	0.005	0.108	0.437
*R*^2^ adj	−0.006	0.084	0.414
Δ*R*^2^	–	0.103	0.329
Robust Wald test for added block	–	5.359***	16.109***
Robust omnibus Wald test	0.657	3.575***	7.745***

*β* = standardised regression coefficient. *p*-values were calculated using heteroscedasticity-consistent. HC3 robust standard errors. *p < 0.05, **p < 0.01; ***p < 0.001.

### 3.4 The time of arrival in Poland on the assessment of intrapartum care and midwifery care

The results of the moderation analyses are presented in Table 4. A significant interaction effect was found between the fulfilment of childbirth-related expectations and the time of arrival in Poland, both for the overall assessment of intrapartum care (*β* = 0.328; *p* = 0.002) and for the assessment of midwifery care (*β* = 0.248; *p* = 0.021). These results indicate that the positive relationship between the fulfilment of childbirth-related expectations and the assessment of care was stronger among women who arrived in Poland before the outbreak of the war. Significant interaction effects were also observed for the experience of discrimination or humiliation (*β* from −0.167 to −0.211; *p* < 0.001), as well as for worse treatment based on national origin (*β* from −0.233 to −0.206; *p* < 0.001) in both models analysed. These negative interpersonal experiences were associated with significantly lower care assessment scores, with this effect being clearly stronger among women who arrived in Poland during the war. No significant interaction effects were found for being informed about the medical situation or for the language barrier with respect to any of the dependent variables analysed (*p* > 0.05), which suggests that the impact of these factors on care assessment did not depend on the time of arrival in Poland.

**Table 4:** Moderation effects of the time of arrival in Poland on the assessment of intrapartum care and midwifery care.

Interaction (X × Moderator)	*β* – Assessment of intrapartum care	*p*-value	*β* – Assessment of midwifery care	*p*-value
Fulfilment of childbirth-related expectations × Arrival in Poland during the war	0.328	0.002	0.248	0.021
Being informed about the medical situation × Arrival in Poland during the war	−0.099	0.364	0.104	0.330
Experience of discrimination/humiliation × Arrival in Poland during the war	−0.167	<0.001	−0.211	<0.001
Experience of worse treatment due to national origin × Arrival in Poland during the war	−0.233	<0.001	−0.206	<0.001
Language barrier × Arrival in Poland during the war	−0.072	0.173	−0.101	0.058

## 4 Discussion

The assessment of women’s childbirth experiences and evaluation of health professionals providing care during labour have long attracted the attention of researchers around the world (16, 25, 26). At the same time, studies on the health of migrant women, including their experiences with perinatal care in host countries, constitute an important area of contemporary scientific analysis, pointing to variation in outcomes and the factors that determine them (15, 27, 28). In Poland, Ukrainian nationals have long represented the largest group of migrants, and after the Russian Federation’s invasion of Ukraine, their number increased substantially, particularly among women (27, 29). In this context, the present study aligns with current research by exploring the opinions of women from Ukraine regarding their childbirth experiences, including their perceptions of midwifery care in Poland.

Due to the growing scale of migration, healthcare personnel in host countries are increasingly providing care to culturally and linguistically diverse patients, which can lead to a range of challenges and difficulties, including those related to communication (17, 18). Issues related to communication and proficiency in the host country’s language constitute important factors shaping how migrant women experience and perceive the healthcare they receive (17, 19). Bains et al. found that poor understanding of the host country’s healthcare system may hinder migrant women’s ability to navigate its structures, including access to perinatal care. These difficulties, together with insufficient proficiency in the host country’s language, psychosocial factors, and differing expectations regarding healthcare, constitute significant challenges and barriers to providing migrant women with optimal care (18). Findings from a study conducted in Switzerland also indicate that a language barrier and insufficient communication regarding the patient’s medical situation are associated with negative experiences of perinatal care. The authors emphasise that effective communication is one of the key elements in understanding one’s health situation, which may directly improve the quality of the care received (20). At the same time, the findings of our study showed that the ability to communicate in Polish was a significant positive factor associated with better assessment of intrapartum care. Furthermore, our study showed that fulfilment of childbirth-related expectations, as well as being informed about the medical situation were significantly associated with more favourable assessments of intrapartum and midwifery care. These findings are broadly consistent with previous reports from Poland and other European countries, indicating that communication barriers, limited understanding of maternity care services, and unmet information needs may be associated with less favourable maternity care experiences among migrant women (17–20, 27, 29). In practice, maternity services should provide clear, linguistically accessible information and routinely discuss women’s childbirth-related expectations. However, the cross-sectional design of the study does not allow the direction of these associations to be determined. It cannot be established whether communication in Polish, information about the medical situation, and fulfilment of childbirth-related expectations influenced women’s evaluations of care, or whether women with more favourable care evaluations were more likely to perceive communication as better and their expectations as fulfilled.

The literature consistently indicates that experiencing discrimination or humiliation is an important negative factor shaping migrant women’s assessments and experiences of maternity care (19, 22, 30). In their systematic review, Fair et al. demonstrated that migrant women receiving maternity care more frequently expressed a sense of being treated differently compared with citizens of the host country, pointing to origin-related stereotyping and prejudice (21). Our findings were consistent with these observations. Experiences of discrimination or humiliation were associated with lower assessments of both intrapartum and midwifery care, whereas worse treatment related to national origin was associated with lower intrapartum care assessment.

Our findings showed that the use of epidural anaesthesia during labour was associated with lower midwifery care assessment scores. These results are in line with reports on women’s overall satisfaction with their perinatal experiences. The literature indicates that epidural analgesia, similarly to other interventions that alter the physiological course of labour, is significantly associated with lower maternal satisfaction with childbirth. At the same time, there is a positive relationship between reduced medicalisation of labour and higher satisfaction with the childbirth experience (31, 32). In contrast, a study from Saudi Arabia found that using epidural analgesia significantly improved women’s satisfaction with their perinatal experiences. Another factor that increased women’s satisfaction was clear and understandable communication about the medical procedures undertaken (33). These differences may reflect variation in clinical indications, women’s expectations, models of care, and the methods used to assess satisfaction or care across studies. In addition, the use of non-pharmacological pain relief methods remained positively correlated with the assessment of midwifery care in the final model, which may be consistent with reports linking less medicalised childbirth practises to more favourable birth experiences (31, 32).

Our moderation analysis indicated that the associations between selected psychosocial variables and assessments of intrapartum care differed according to the timing of migrant women’s arrival in Poland in the context of war-related migration from Ukraine. These findings suggest that childbirth experiences and interactions with the healthcare system are not homogeneous within the population of migrant women but are deeply embedded in broader social, emotional, and migratory contexts. The available literature also confirms that the population of migrant women is highly heterogeneous. A 2024 study from Norway found that refugee women were more likely to experience differences in treatment related to their religion, language, or skin colour compared with migrant women who had not fled war. Furthermore, the authors noted that these women were less likely to understand the information provided by healthcare staff (34). Other studies also confirm the heterogeneity of the migrant population, considering such factors as the region of origin and the recency of migration. These factors were associated with differences in women’s assessments of maternity care. Recent migrant women, especially those from EU accession countries, were less likely to report that communication with medical staff helped them understand their health situation. Moreover, they stated that they were not treated with kindness at every stage of care (35).

In our study, the strongest moderating effect was observed for the fulfilment of childbirth-related expectations, which was associated with a more positive assessment of both intrapartum and midwifery care among women who arrived in Poland before the outbreak of the war. This may suggest that, within this group of migrant women, expectations regarding care were more clearly formed and more realistic, stemming from their longer experience of navigating the Polish healthcare system. For migrant women who arrived in Poland during the war, childbirth may have been a situational event resulting from necessity, potentially reducing the relevance of the subjective alignment of care with the women’s prior expectations. Available reports indicate an association between unfulfilled childbirth-related expectations and lower satisfaction with the birth experience. However, these findings pertain to the general population rather than migrant women (36, 37). The lack of studies examining the relationships between expectations, length of stay, and satisfaction with perinatal care among migrant women limits the possibility of an in-depth interpretation of the results obtained in this study.

In their 2025 systematic review, Arcilla et al. analysed racial discrimination in maternal care experienced by migrant women. None of the studies included in the review originally aimed to analyse experiences of discrimination, as their primary focus was the overall assessment of maternal care quality. Nevertheless, the findings revealed that experiences of discrimination were widespread among the migrant women studied (23). Migrant women reporting mistreatment during maternal-newborn care most frequently indicated verbal abuse, stigmatisation, criticism, and a lack of respect for their religious and cultural needs from healthcare staff. Despite experiencing negative events, some women still viewed the overall care positively, as confirmed by findings from a study conducted in Italy (24). In turn, a study conducted in Poland in 2023 among migrant women from Ukraine indicates that they faced numerous barriers in accessing maternity care, including concerns about discrimination. Migrant women permanently settled in Poland, as well as those regularly travelling between Poland and Ukraine proved to be a particularly vulnerable group, as they more often reported increasing hostility from people around them (27). At the same time, our findings demonstrated that negative experiences related to discrimination, humiliation, and a sense of being treated worse due to national origin significantly lowered the assessment scores for intrapartum care among women who arrived in Poland during the war. The increased sensitivity of this group of migrant women to exclusion and poorer treatment may result from the accumulation of stress associated with forced migration, marked by traumatic experiences, uncertainty, and reduced access to social support. This can have a disproportionately strong impact on the assessment of intrapartum care, even if it stems from just one negative encounter with medical staff. Furthermore, it should be emphasised that the cross-sectional design of the study does not allow conclusions to be drawn regarding the direction or mechanisms of these associations. These findings underscore the need for respectful and culturally responsive perinatal care, staff training in intercultural communication, and accessible mechanisms for reporting and addressing discrimination-related concerns.

Our moderation analysis showed that the associations between selected psychosocial variables and ratings of intrapartum care varied according to the timing of migrant women’s arrival in Poland. These findings suggest that childbirth experiences and interactions with the healthcare system may differ across migrant populations depending on broader social, emotional, and migration-related contexts. This is consistent with previous evidence highlighting the importance of effective communication and proficiency in the language of the host country (34, 38–41).

Overall, the observed associations differed according to the timing of arrival in Poland, highlighting the need to consider the migration context and duration when designing communication, support, and perinatal care strategies for women affected by war-related migration.

## 5 Limitations

The present study allowed for analysing the childbirth experiences of 481 migrant women from Ukraine who gave birth in Poland. This represents a major strength of our study, as it enables the identification of factors influencing the quality of care provided during childbirth. Furthermore, the study considered the migration context created by the outbreak of the full-scale war in Ukraine, which facilitated a better understanding of the specific needs of these women and pointed to aspects of the healthcare system that still require improvement. However, our study has certain limitations. First, it was conducted using an online survey technique, and the data relied on participants’ self reports, which made it impossible to verify clinical information. Furthermore, participation in the study was voluntary and limited to individuals using social media. This means that women who were not active online, as well as those for whom the topic was not considered important or relevant, may not have taken part in the study. The original questionnaire was developed specifically for the purposes of this study and was pilot tested; however, it did not undergo formal psychometric validation. Its content validity, internal consistency, and reliability were not assessed. Therefore, the findings should be interpreted cautiously, as a description of participants’ subjective experiences rather than as measurements obtained using a fully validated instrument. The use of single numerical ratings enabled a global assessment of intrapartum and midwifery care but may not have fully captured the multidimensional nature of childbirth experiences. Future studies should incorporate culturally adapted and validated instruments to more comprehensively assess specific domains of perinatal care in Poland from the perspective of migrant women. Furthermore, the subjective nature of childbirth experiences and the varying amount of time that had passed since delivery may have influenced how these experiences were perceived and reported. The cross-sectional design precludes causal inference; therefore, the observed associations should be interpreted as relationships rather than causal effects. In addition, other components of perinatal care, such as antenatal care, and the contributions of other members of the interdisciplinary care team were not assessed. Despite these limitations, the findings provide valuable insights into the experiences of migrant women receiving intrapartum and midwifery care in Poland. Further research in this area is necessary to deepen understanding of the needs of migrant and refugee women and to develop solutions that ensure adequate and culturally sensitive perinatal care tailored to their needs.

## 6 Conclusion

The findings suggest that psychosocial and communication-related aspects of childbirth care-particularly fulfilment of childbirth-related expectations, adequate information, and equal treatment-were more strongly associated with positive assessments of intrapartum and midwifery care than clinical characteristics of labour. These results highlight the importance of respectful, patient-centred, and culturally responsive maternity care for Ukrainian migrant women in Poland. In addition, the migration context related to the war appeared to modify the relationship between selected psychosocial factors and care evaluations, indicating the need for tailored support strategies for different migrant subgroups.

## Data Availability

The original contributions presented in the study are included in the article/supplementary material, further inquiries can be directed to the corresponding author.
